# Methylphenidate-Induced Non-ischemic Heart Failure With Reduced Ejection Fraction and Mild Pulmonary Hypertension

**DOI:** 10.7759/cureus.55604

**Published:** 2024-03-05

**Authors:** Derek Ugwendum, Yolande Mbome, Divine Besong Arrey Agbor, Umida Burkhanova, Rita Offor, Ikpechukwu J Okorie, Asher Gorantla, Frances A Amokaye, Muhammed Atere, Jay Nfonoyim

**Affiliations:** 1 Internal Medicine, Richmond University Medical Center, Staten Island, USA; 2 Cardiology, State University of New York (SUNY) Downstate Medical Center, Brooklyn, USA; 3 Internal Medicine, State University of New York (SUNY) Downstate Medical Center, Brooklyn, USA; 4 Internal Medicine, American University of Antigua, St. John's, ATG; 5 Pulmonary and Critical Care, Richmond University Medical Center, Staten Island, USA

**Keywords:** implantable cardioverter defibrillator (icd), central nervous system stimulant, heart failure with reduced ejection fraction, methylphenidate, attention deficit hyperactivity disorder (adhd)

## Abstract

Attention-deficit hyperactivity disorder (ADHD) is a neurodevelopmental disorder that is commonly diagnosed during childhood. Patients present with hyperactive-impulsive behavior and/or inappropriate inattention which may persist through adulthood.

Central nervous system stimulants have been used to manage patients with ADHD. Methylphenidate which is used as a first-line therapy has been shown to have adverse cardiovascular effects in these patients. This is a case of a young male with a history of ADHD since childhood on methylphenidate who was diagnosed with acute non-ischemic heart failure with an ejection fraction of 15-20%. Methylphenidate-induced heart failure is the rare adverse effect seen in ADHD patients who are on this medication. Our patient was started on goal-directed medical therapy for heart failure and was discharged with an implantable cardioverter defibrillator (LifeVest®, ZOLL, Pittsburgh, PA) because of his persistently low left ventricular ejection fraction.

It is important for physicians to always consider heart failure as a possible cardiovascular adverse effect when starting patients on methylphenidate for the management of ADHD.

## Introduction

Cardiovascular adverse effects that have been commonly associated with methylphenidate therapy includes arrhythmias, myocardial infarction, and hypertension [1,2]. Very few cases have been reported of heart failure induced by methylphenidate [3]. Our case is one of those rare cases who presented with shortness of breath and diagnosed with acute non-ischemic heart failure with an ejection fraction of 15-20% and mild pulmonary hypertension.

## Case presentation

A 33-year-old African American male with a past medical history of attention-deficit hyperactivity disorder (ADHD), diagnosed since childhood, on methylphenidate (Concerta®), with no history of illicit drug use presented to the emergency department (ED) with complaints of worsening shortness of breath, associated with cough productive of blood-tinged phlegm. His symptoms had been ongoing for two weeks and were exacerbated by exertion and laying supine. He denied fever, chills, night sweats, chest pain, weight loss, lower extremity edema, sick contacts, or recent travel. 

Physical examination revealed an alert and oriented patient with no apparent signs of respiratory distress. His vital signs at presentation were BP 120/83 mmHg, respiratory rate 20 breaths per minute, pulse 93 beats per minute, temperature 97.4 F, and oxygen saturation 95% on a 2L nasal cannula. His height and weight were 172.7 cm and 77.8 kg respectively, with a BMI of 26.1 kg/m².

A cardiovascular examination revealed irregular pulses and tachycardia but no murmurs, gallops, or rubs. No jugular vein distention (JVD) was noted. Respiratory examination revealed bibasilar crackles with no use of accessory muscles for breathing. No lower extremity edema was observed. His hematology and chemistry panel were within normal limits. Serum thyroid-stimulating hormone (TSH) of 1.423 uIU/mL, triglycerides of 121 mg/dL, very low-density lipoprotein (VLDL) of 63 mg/dL, cholesterol 158 mg/dL, glycosylated hemoglobin (HbA1C) of 4.8%, high sensitive troponin of 39.1 ng/L, BNP 1052.0 pg/mL. Inflammatory markers (ESR, CRP) were all negative, and blood electrolytes were within normal limits.

**Figure 1:**
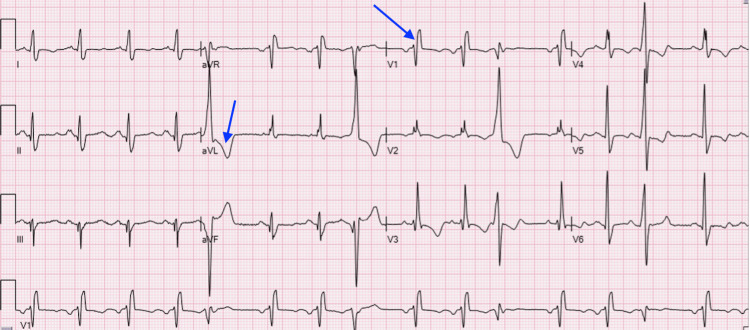
EKG showing sinus rhythm with occasional premature ventricular complexes. Possible left atrial enlargement and right bundle branch block.

Initial EKG revealed sinus rhythm with occasional premature ventricular complexes, and right bundle branch block (Figure 1).

**Figure 2:**
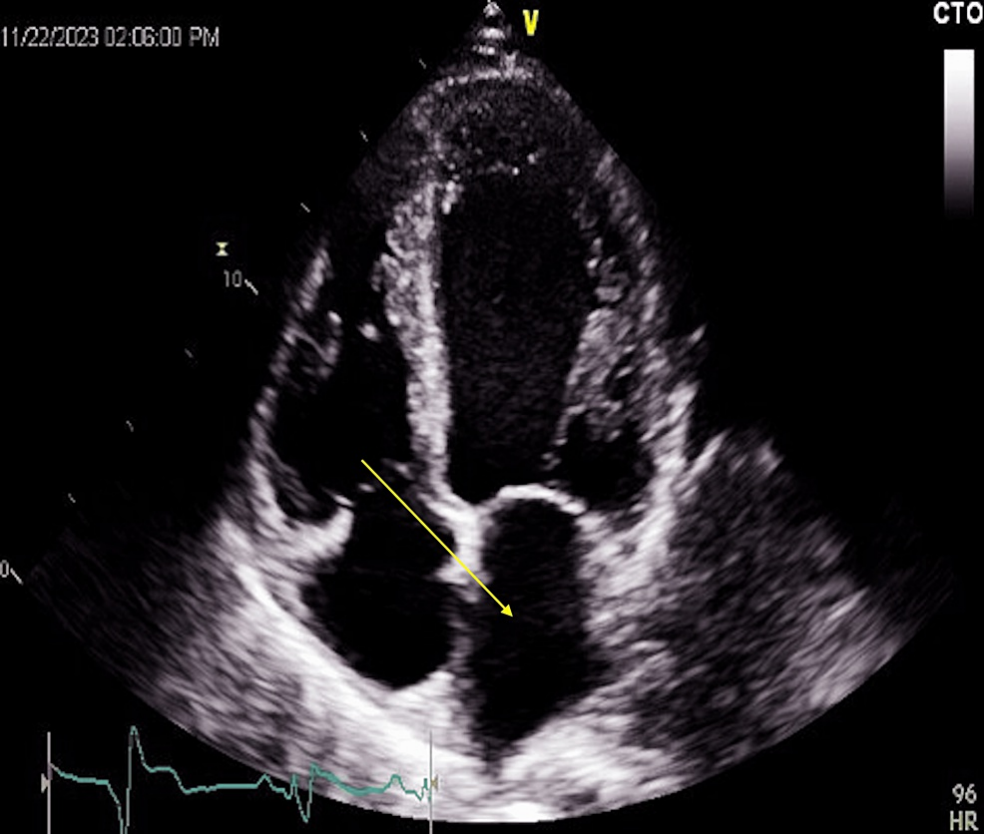
Echography showing a moderately dilated left atrium

His chest X-ray showed mid to lower lung zone mixed interstitial/alveolar infiltrates which may represent interstitial edema. The patient’s echocardiogram revealed severe left ventricular global hypokinesis with a left ventricular ejection fraction of 15-20% with a moderately dilated left atrium, a right ventricular systolic pressure of 40 mmHg, moderate mitral and tricuspid valve regurgitations. There was no eccentric or concentric left ventricular hypertrophy. These findings were consistent with very severe left ventricular systolic dysfunction and a likelihood of mild pulmonary hypertension (Figure 2).

A computed tomography (CT) of the chest showed perihilar alveolar and interstitial pulmonary edema with borderline cardiomegaly consistent with congestive heart failure.

A diagnosis of new-onset heart failure with reduced ejection fraction (HFrEF) was made and he was admitted to the coronary care unit. An interventional cardiologist and electrophysiologist were consulted. The patient was kept NPO (nothing by mouth) for a cardiac catheterization the following day. He was started on furosemide 40 mg once daily intravenously.

**Figure 3:**
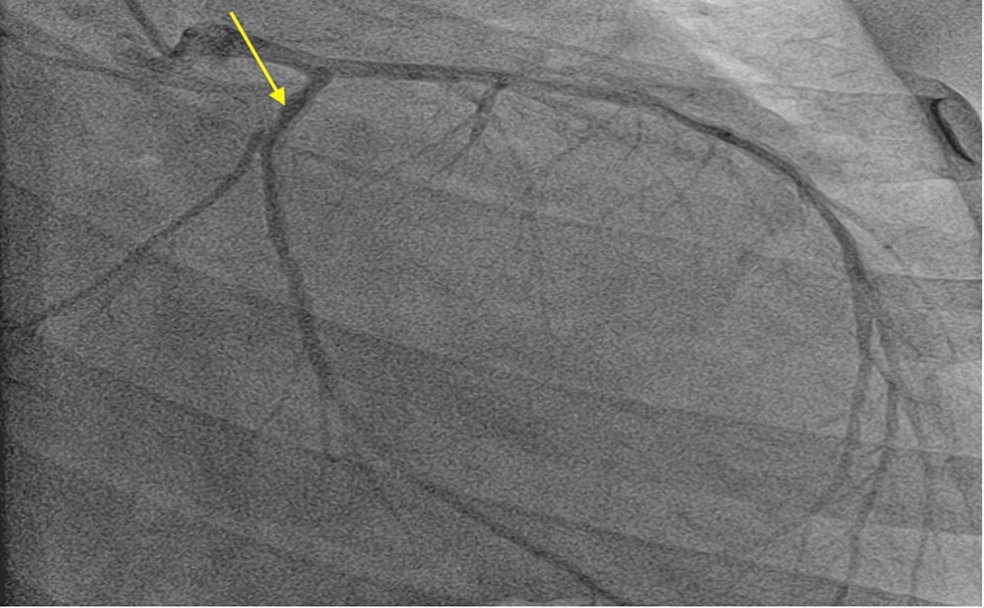
Coronary angiography showing non-occluded coronary arteries

On day 2 of hospitalization, cardiac catheterization was done, and it confirmed normal patency of the coronary arteries and non-ischemic cardiomyopathy as shown in Figure 3 below. Cardiology recommended patient be started on Entresto 24-26 mg 1 tab orally twice daily, metoprolol 25 mg orally daily, and dapagliflozin 10 mg by mouth twice a day.

The electrophysiologist recommended a LifeVest® (ZOLL, Pittsburgh, PA) due to his poor left ventricular ejection fraction of less than 35% and an outpatient follow-up visit within a month of discharge. He was advised to adhere to a low sodium diet and fluid restriction of 1.5-2 L/day.

He was seen on outpatient clinic and a repeat echocardiography was done after three months while being on goal directed medical therapy showed a left ventricular ejection fraction of about 10-15%. He was scheduled for an implantable cardioverter defibrillator (ICD) placement procedure from which he benefitted.

## Discussion

ADHD is a neuropsychiatric disorder that is commonly diagnosed in childhood with a global prevalence rate of 5% among school-age children [4].

ADHD is characterized by a pervasive pattern of impaired attention, hyperactivity-impulsivity behavior, or both [5]. The etiology of ADHD is not well understood [6].

Medications used to treat patients with ADHD have been shown to be effective in reducing symptoms of hyperactivity and impulsivity [7]. These medications act as an agonist of the sympathetic nervous system causing an increase in heart rate and blood pressure through their noradrenergic and dopaminergic effects [8]. Our patient was on methylphenidate 36 mg daily since childhood, a first-line treatment for ADHD.

There have been cardiovascular adverse effects reported with the use of methylphenidate [9]. Some studies have shown an increased risk of arrhythmias and myocardial infarction after starting patients on methylphenidate for the treatment of ADHD [1]. Few case reports about the use of methylphenidate and life-threatening heart failure have been reported in Sweden [3]. Hypertension has been seen in children and adolescents [2]. Cardiac arrest is another reported adverse effect but some studies showed no statistical significance between medications used in ADHD and cardiac arrest [10].

Our patient was a young African male diagnosed with heart failure with reduced ejection fraction with no contributory cardiovascular risk factors such as diabetes, hypertension, and hyperlipidemia. Methylphenidate was discontinued as it was the most probable cause of his heart failure given this medication has cardiovascular adverse effects. He was started on furosemide 40 mg, Entresto 24-26 mg 1 tablet every 12 hours, metoprolol succinate 25 mg 1 tablet daily, and dapagliflozin 10 mg 1 tablet every 12 hours for the management of HFrEF. He was discharged with a LifeVest®. Despite being on goal-directed medical therapy (GDMT), his repeat echocardiography showed a left ventricular ejection fraction of about 10-15%. This may suggest an irreversible cardiomyopathy caused by methylphenidate therapy. An automated implantable cardioverter defibrillator was placed due to a lack of improvement to his left ventricular ejection fraction despite compliance with GDMT.

## Conclusions

Methylphenidate which is considered first-line treatment for attention-deficit hyperactivity disorder (ADHD) has been reported to have adverse cardiovascular effects. Few case reports of heart failure have been reported in patients with ADHD on methylphenidate. Our case is one of those rare cases of methylphenidate-induced cardiomyopathy as the most probable cause. His inflammatory markers to investigate for viral and inflammatory etiologies were negative. Echocardiography did not show findings typical of an infiltrative cardiomyopathy. It is worth noting that this may be a potential adverse effect of this medication. Increased physician awareness of this adverse effect, especially when a patient starts presenting with symptoms such as shortness of breath, will improve patient surveyance and reduce the incidence of a more tragic cardiovascular outcome.
